# UGT-YOLO: A Multi-Strategy Fusion Model for Automated Dairy Cow Body Condition Scoring

**DOI:** 10.3390/ani16162522

**Published:** 2026-08-12

**Authors:** Ye Wang, Xin-Ning Wang, Hong-Rui Guo, Zhi-Xin Gu

**Affiliations:** 1 College of Control and Information Engineering, Northeast Forestry University, Harbin 150040, China; 2 College of Electronic Science and Engineering, Jilin University, Changchun 130012, China

**Keywords:** body condition scoring, dairy cattle, deep learning, feature fusion, real-time detection, YOLOv11

## Abstract

Dairy cow body condition score (BCS) is a practical, semi-quantitative indicator of body energy reserves and changes in energy balance. To improve five-class BCS detection under complex imaging conditions, this study developed UGT-YOLO by integrating a UniRepLKNet Block, a Gather-and-Distribute feature-fusion mechanism, and a Task-Aligned Dynamic Detection Head into YOLOv11n. The model was evaluated using a single publicly available dataset containing five adjacent BCS classes: 3.25, 3.50, 3.75, 4.00, and 4.25. Following redundancy removal and image-quality screening, 7015 original images were retained. Dataset partitioning was completed before data augmentation. The original images were divided at the source-video-sequence level into training, validation, and test subsets containing 5612, 702, and 701 images, respectively. Available cow identifiers were additionally used to keep images of the same identified animal within a single subset. Data augmentation was applied exclusively to the training subset, increasing the training set to 9639 images and producing a final experimental dataset of 11,042 images. UGT-YOLO achieved a precision of 84.3%, a recall of 81.1%, an mAP@0.5 of 88.5%, and an mAP@0.5:0.95 of 66.8%. Compared with YOLOv11n, these values increased by 4.8, 0.9, 2.8, and 4.5 percentage points, respectively. The parameter count increased from 2.6 to 6.5 million, and computational cost increased from 6.4 to 19.2 GFLOPs. Under an input resolution of 640 × 640 pixels, a batch size of 1, and FP32 inference on an NVIDIA GeForce RTX 3090, UGT-YOLO achieved a throughput of 68 frames s^−1^. These results demonstrate an accuracy–complexity trade-off within the evaluated public dataset and restricted BCS range. Independent cow-level, cross-farm, full-range BCS, multi-scorer, and edge-device validation remains necessary.

## Simple Summary

Automated, non-contact assessment of dairy cow body condition can provide more frequent, consistent, and repeatable information for feeding and health-management decisions. In this study, the proposed vision model was evaluated using a single publicly available image dataset containing five adjacent body condition score classes ranging from 3.25 to 4.25. Compared with the baseline YOLOv11n model, UGT-YOLO improved five-class detection performance while retaining real-time image-level inference on an NVIDIA GeForce RTX 3090 under the reported benchmark conditions. Because the study did not include cows outside this score range or an independent farm dataset, further cow-level, cross-farm, full-range BCS, and edge-device validation is required before practical deployment.

## 1. Introduction

Body condition score (BCS) is a widely used, non-invasive and semi-quantitative indicator of body energy reserves in dairy cows. It is assessed from the prominence of skeletal structures and the extent of subcutaneous fat coverage at anatomical regions such as the tailhead, hook bones, pin bones, lumbar transverse processes, and spine [1]. Changes in BCS across the production cycle provide useful information on the relationship between energy intake and energy requirements and have been associated with subsequent health, milk production, and reproductive outcomes [2,3]. However, BCS does not directly measure metabolic status and should be interpreted together with physiological, production, and clinical indicators.

Excessive loss of body condition during the transition period and early lactation is associated with increased mobilisation of adipose reserves and elevated circulating concentrations of non-esterified fatty acids and β-hydroxybutyrate. These changes are recognised risk indicators for metabolic disorders such as ketosis and fatty liver, although their clinical significance depends on initial BCS, parity, lactation stage, milk yield, and the timing and rate of condition loss [4,5]. Excessive body condition before calving has likewise been associated with an increased risk of postpartum metabolic disorders.

For a long time, the assessment of dairy cow body condition has mainly relied on regular visual observation and physical palpation performed by experienced livestock veterinarians or nutritionists. The most internationally widespread and influential system was established by Wildman et al. [6], with a grading interval of 0.25 points on a 1-to-5 scale. This system requires the scorer to comprehensively evaluate the fat cover thickness and bone prominence of key anatomical sites including the tailhead, hip bones (tuber coxae), pin bones (tuber ischii), transverse processes of the lumbar vertebrae, and spine [7].

Manual BCS assessment is commonly based on visual observation and may be supplemented by palpation of selected anatomical regions. Visual assessment can generally be performed without direct animal handling and is therefore considered a low-disturbance procedure. Its principal limitations are scorer subjectivity, inter- and intra-observer variability, visual fatigue, labour requirements, and limited measurement frequency. Palpation or close physical inspection may cause brief disturbance in some management settings, but it is not an inherent requirement of routine visual BCS assessment. Automated image-based assessment may improve scoring frequency and consistency while reducing dependence on repeated manual observation.

Leveraging the performance of deep learning in image recognition, features of the back and hindquarters of dairy cows can be captured using high-definition cameras during milking or walking. This approach enables non-contact data collection and supports automatic scoring based on learned visual features, thereby reducing evaluator-dependent variability [8].

Early automated BCS studies mainly relied on manually designed morphological features extracted from two-dimensional images. These approaches quantified body contours, angular characteristics, skeletal prominence and concavity around the hindquarter region, followed by statistical or conventional machine-learning models for BCS estimation [9,10,11]. Related work also applied machine learning to morphological measurements for automated dairy cattle phenotyping [12]. Although two-dimensional imaging is relatively inexpensive and easy to implement, its performance depends strongly on image segmentation and handcrafted feature selection and is therefore sensitive to coat patterns, illumination changes, posture variation and background interference. To obtain more explicit geometric information, subsequent studies introduced Kinect sensors, depth cameras, three-dimensional point clouds and multi-camera systems [13,14,15,16,17,18,19]. Depth-image CNNs, continuous three-dimensional monitoring and ensemble models further demonstrated the value of richer geometric representations, although their wider application remains restricted by sensor cost, calibration requirements, installation conditions and the computational complexity of three-dimensional data processing.

With the development of deep learning, automated BCS assessment has gradually shifted from handcrafted feature engineering to end-to-end feature learning. Huang et al. [20] demonstrated that a deep learning detector could localise BCS-related regions under complex backgrounds, while Li [21] compared multiple detection architectures for image-based BCS assessment. Sun et al. [22] further combined individual identification from body-part features with automated BCS estimation in a unified deep learning framework. More recent studies have increasingly focused on two-dimensional RGB imaging because conventional cameras are less expensive and easier to integrate into existing farm infrastructure. Siachos et al. [23] developed and validated a fully automated two-dimensional imaging system using separate training and testing datasets and evaluated agreement with trained human assessors. Mullins et al. [24] validated a commercial automated BCS system on a commercial dairy farm and highlighted reduced accuracy at under- and over-conditioned extremes. Zheng et al. [25] proposed BCS-YOLO by combining lightweight structural optimisation with knowledge distillation. Li et al. [26] extended image-level BCS prediction to continuous video processing and herd-level body-condition distribution analysis. Lewis et al. [27] compared front, rear and top-down views and found that rear-view images provided the most informative representation of BCS-related anatomical features. Liu et al. [28] further explored lightweight five-class BCS assessment for edge-oriented deployment.

Collectively, these studies demonstrate that automated BCS can be approached through low-cost two-dimensional imaging, geometrically informative three-dimensional sensing, image-level detection, ordinal prediction, individual identification and continuous video analysis. However, reported performance varies substantially because of differences in scoring ranges, imaging viewpoints, dataset composition, independence of training and test samples, use of knowledge distillation or pretraining, and evaluation metrics. Recent three-dimensional point-cloud work, for example, reported exact and tolerance-based accuracies rather than object-detection mAP; depth-image ensemble models similarly emphasised tolerance-based scoring, and continuous three-dimensional or commercial systems prioritised on-farm monitoring and agreement with manual assessment. Multi-farm two-dimensional systems also emphasised repeatability and agreement with human scorers. Consequently, numerical results from different studies should not be interpreted as directly interchangeable.

An additional challenge is the uncertainty associated with adjacent quarter-point BCS categories. Morphological differences between categories such as 3.25 and 3.50 may be subtle and can be affected by posture, illumination, coat pattern, viewing angle, and the scorer’s interpretation of anatomical landmarks. Disagreement among trained assessors is therefore possible, particularly for animals located near a class boundary. In the present study, the expert-assigned labels supplied with the public dataset were treated as the reference standard. However, repeated scoring records and inter-rater agreement statistics were unavailable. Consequently, some apparent disagreements between model predictions and reference labels may reflect uncertainty at adjacent class boundaries rather than unequivocal model failure.

Despite this progress, several challenges remain. First, adjacent quarter-point BCS categories, such as 3.25 and 3.50, exhibit substantial morphological overlap, and subtle differences in fat coverage around the tailhead, hip bones and pin bones may be weakened during feature extraction and multi-scale transmission. Second, conventional feature-pyramid structures mainly transmit information between neighbouring layers, potentially limiting long-range cross-level interaction and causing loss of fine morphological details. Third, classification confidence and localisation quality may remain spatially inconsistent in conventional detection heads. In addition, improvements in detection accuracy are frequently accompanied by increased computational cost, while evidence from independent farms and edge devices remains limited. Further investigation is therefore required to improve fine-grained BCS detection while maintaining practical image-level inference efficiency.

Accordingly, this study developed UGT-YOLO based on YOLOv11n for fine-grained dairy cow BCS detection. UniRepLKNet Block was incorporated into the backbone to enlarge the effective receptive field and strengthen global morphological representation. The Gather-and-Distribute mechanism was introduced into the neck to improve cross-level feature interaction, while a Task-Aligned Dynamic Detection Head was adopted to coordinate classification and localisation. The objectives were to: (1) evaluate whether the three complementary modifications improve five-class BCS detection; (2) quantify their individual and combined contributions through comparative and ablation experiments; and (3) assess the accuracy-complexity trade-off and the potential of the model for real-time image-level monitoring.

## 2. Materials and Methods

### 2.1. Dataset Source and Study Design

#### 2.1.1. Source Dataset and Annotation

This study used the publicly available “Dairy cow body condition score target detection data set” released by Huang et al. [29]. According to the source documentation, the complete dataset contains 53,566 images obtained from 5704 dairy cows between May 2018 and July 2023 at three recording locations. The original category distribution comprised 7536 images for BCS 3.25, 13,256 images for BCS 3.50, 14,255 images for BCS 3.75, 12,556 images for BCS 4.00, and 5963 images for BCS 4.25. Images were extracted from MP4 videos recorded using a Hikvision DS-2CD3T56 DWD-I5 (Hangzhou Hikvision Digital Technology Co., Ltd., Hangzhou, China) two-dimensional network camera with a nominal resolution of five megapixels. The source videos had a resolution of 1297 × 720 pixels, and the released images were provided at 1024 × 576 pixels. The tailhead and hindquarter regions were manually annotated using LabelImg (version 1.8.6), and bounding-box annotations were stored in PASCAL VOC XML format. BCS labels were assigned by an experienced animal-husbandry and veterinary team. The source documentation reported three recording locations but did not explicitly state whether these locations represented three independently managed farms. The total number of distinct farms and the total number of source video files were therefore considered unavailable. The source documentation did not report the exact number of annotators, whether BCS labels were independently assigned by multiple scorers, how disagreements were resolved, or whether inter-rater agreement was assessed. These details were therefore considered unavailable.

#### 2.1.2. BCS Categories and Image Characteristics

**Figure 1:**
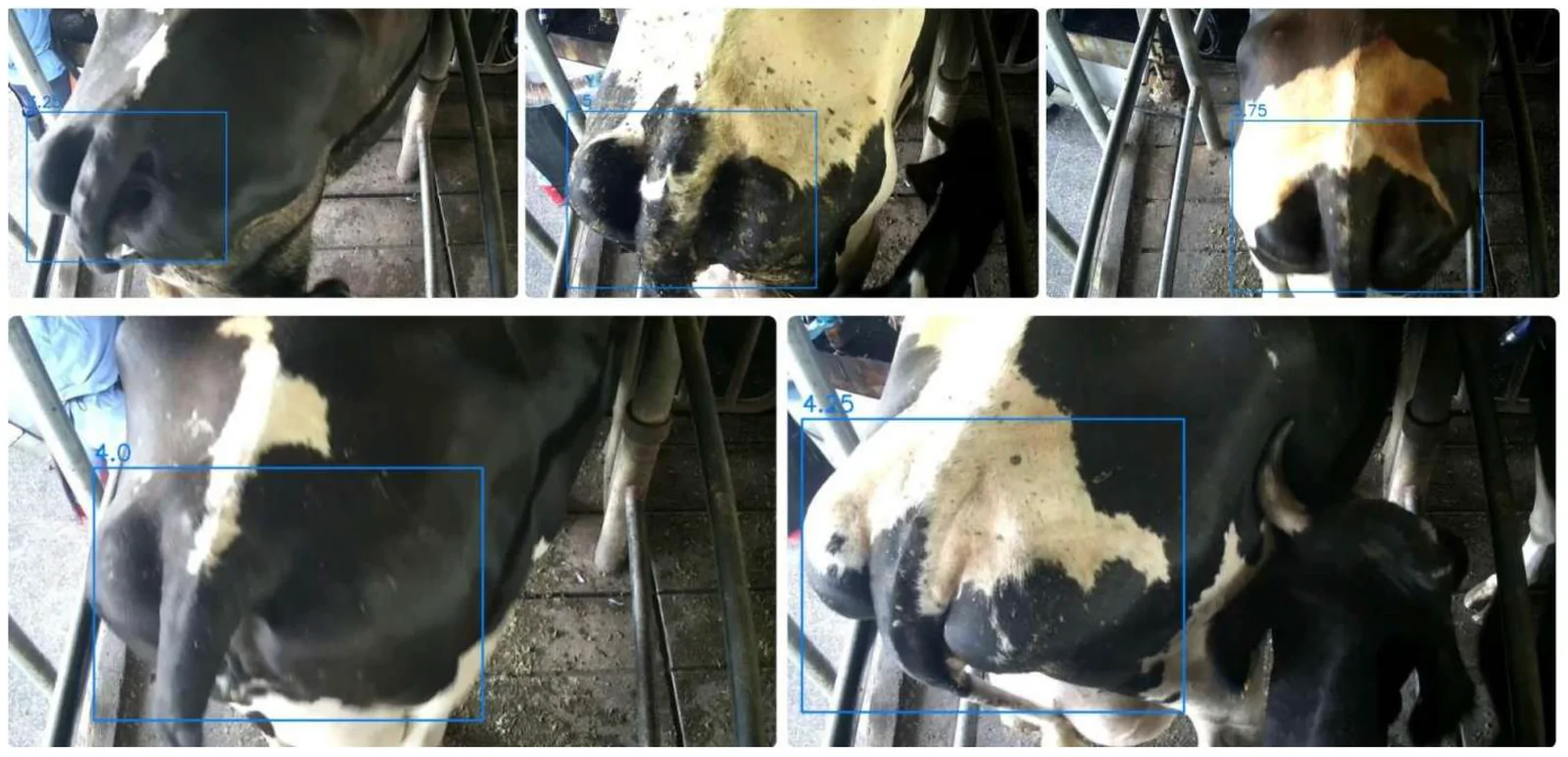
Representative annotated tailhead and hindquarter images from the five BCS categories.

Conventional dairy cow BCS systems commonly use a scale ranging from 1 to 5, frequently divided into increments of 0.25. However, the dataset adopted in this study contained only five adjacent quarter-point categories: 3.25, 3.50, 3.75, 4.00, and 4.25. Representative annotated images from these five BCS categories are shown in Figure 1. Therefore, the present study represents a five-class object-detection task within a restricted BCS interval rather than an evaluation across the complete 1–5 scale.

The images primarily depict the tailhead and hindquarter regions, including the tailhead, hook bones, pin bones, and part of the sacral and lumbar regions. These anatomical structures provide visual information related to skeletal prominence and subcutaneous fat coverage. Because images with BCS values below 3.25 or above 4.25 were not included in the source dataset, model performance cannot be extrapolated beyond the evaluated range.

### 2.2. Data Preprocessing

#### 2.2.1. Redundancy Removal and Key-Frame Selection

The source dataset contained numerous temporally adjacent frames acquired from fixed camera positions. Images were first grouped according to source video sequence and arranged in temporal order. A 64-bit perceptual hash was calculated for each frame after conversion to greyscale and resizing to 32 × 32 pixels. A discrete cosine transform was applied, and the 8 × 8 low-frequency coefficients were binarised relative to their median value. Consecutive frames with a perceptual-hash Hamming distance of five or less were treated as candidate near-duplicates. This Hamming-distance criterion was used as a permissive preliminary filter; final frame removal was determined using SSIM to reduce the risk of discarding frames containing meaningful morphological or postural changes.

Candidate frame pairs were subsequently compared using the structural similarity index measure after greyscale conversion and resizing to 256 × 256 pixels. SSIM was calculated using an 11 × 11 Gaussian window, with $K_{1}=0.01$, $K_{2}=0.03$, and a pixel dynamic range of 255. When the SSIM value of two consecutive candidate frames exceeded 0.85, only one representative image was retained. The image with the greater variance of the Laplacian was preferentially retained because it exhibited greater edge sharpness. Images with severe motion blur, substantial occlusion, incomplete target regions, or invalid bounding-box annotations were additionally removed.

The SSIM threshold was selected through a preliminary sensitivity analysis using thresholds of 0.80, 0.85, 0.90, and 0.95. Twenty randomly selected video sequences were examined, and 100 candidate frame pairs were manually evaluated at each threshold. A threshold of 0.85 provided the most appropriate balance between removing visually redundant frames and retaining meaningful changes in posture, illumination, and anatomical appearance. Following redundancy removal, image-quality screening, and class-balancing procedures, 7015 original images were retained.

#### 2.2.2. Dataset Partitioning

Dataset partitioning was completed before any offline or online data augmentation. The 7015 cleaned original images were divided into training, validation, and test subsets using a stratified group-splitting procedure. Source-sequence identifiers were reconstructed from image filename prefixes and consecutive frame numbering. Stratified group splitting was performed using random seed 42. Source video sequence was used as the primary grouping variable, and all images originating from the same continuous video sequence were assigned exclusively to one subset. When multiple sequences had a reliable common cow identifier, those sequences were assigned to the same subset. This procedure prevented temporally adjacent frames and images associated with the same available cow identifier from occurring across the training, validation, and test subsets.

The training, validation, and test subsets contained 5612, 702, and 701 original images, respectively, corresponding approximately to an 8:1:1 ratio. No source video sequence occurred in more than one subset, and no available cow identifier crossed subset boundaries. Group independence was prioritised over obtaining an exact image-level ratio. Because complete cow identifiers were unavailable for all source sequences, sequence-level independence was fully enforced, whereas cow-level independence could be verified only for sequences with available cow identifiers.

#### 2.2.3. Training-Set Augmentation

Data augmentation was applied exclusively to the training subset after dataset partitioning. Offline augmentation included horizontal flipping, image scaling, brightness adjustment, contrast adjustment, saturation variation, colour jitter, Gaussian noise, and histogram equalisation. Bounding-box coordinates were transformed simultaneously with the corresponding images. A total of 4027 offline augmented images were generated, increasing the training set from 5612 original images to 9639 images.

**Figure 2:**
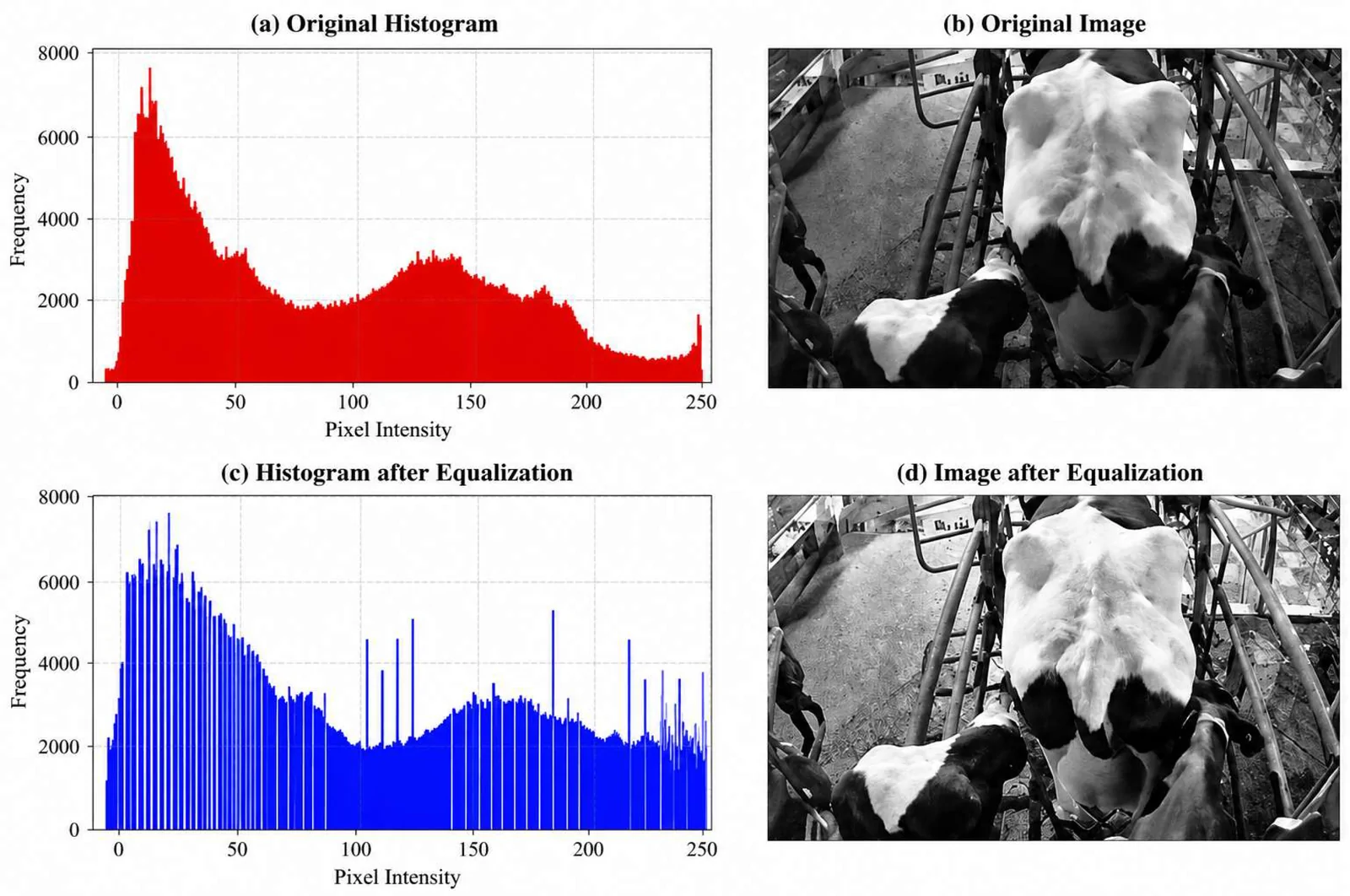
Comparison of histogram equalisation data augmentation effects.

The validation and test subsets were not augmented and contained 702 and 701 original images, respectively. Consequently, the final experimental dataset comprised 11,042 images. Mixup and Mosaic augmentation were applied online only during model training, and Mosaic augmentation was disabled during the final 10 epochs. The validation set was used for checkpoint selection, whereas the test set was evaluated only after model development and checkpoint selection had been completed. Representative results of histogram-equalisation augmentation are shown in Figure 2.

Table 1 summarises the class distribution before and after offline training-set augmentation. Dataset partitioning was performed using only the 7015 cleaned original images. Offline augmentation was subsequently applied exclusively to the training subset. The validation and test subsets contained only original images and were not augmented. Mosaic and MixUp generated stochastic training inputs online and were therefore not counted as additional fixed images in Table 1.

### 2.3. UGT-YOLO Architecture

#### 2.3.1. Overall Architecture

To address the limited receptive field of the baseline model when extracting large-scale anatomical features such as the back, ribs and tailhead, UniRepLKNet Block [30] was incorporated into the deep feature-extraction stage of the backbone. The larger effective receptive field is intended to capture contextual information across the cow torso and to reduce fragmentation of global morphological cues.

In the feature-fusion stage, conventional FPN and PANet structures mainly exchange information through adjacent levels. The Gather-and-Distribute mechanism, originally proposed in Gold-YOLO [31], was introduced to aggregate multi-scale features and redistribute the integrated information to different branches. This design is intended to strengthen interaction between shallow visual details and deeper semantic representations under complex backgrounds.

**Figure 3:**
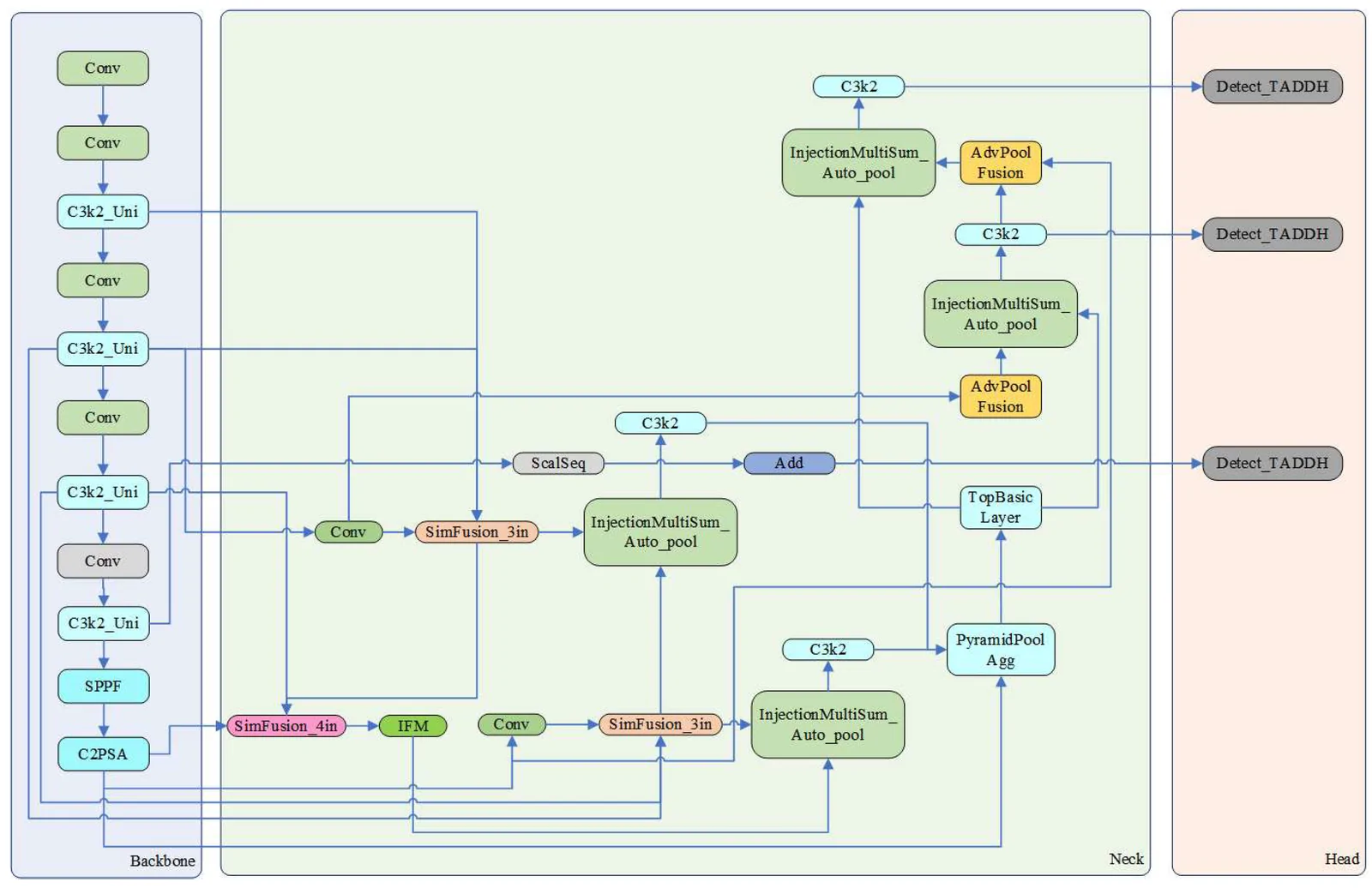
Overall architecture diagram.

To address differences in the feature requirements of classification and regression, a Task-Aligned Dynamic Detection Head was adopted. The task-alignment strategy follows the principle of Task-Aligned One-Stage Object Detection [32], while dynamic spatial and channel reweighting is informed by Dynamic Head [33]. The design seeks to improve consistency between classification confidence and localisation quality while retaining task-specific prediction branches. The proposed UGT-YOLO architecture is shown in Figure 3.

For an input resolution of 640 × 640 pixels, UGT-YOLO generated three prediction feature levels: P3/8, P4/16, and P5/32, corresponding to spatial resolutions of 80 × 80, 40 × 40, and 20 × 20 pixels, respectively. The corresponding output-channel dimensions were 64, 128, and 256. The original C3k2 modules at the P3, P4, and P5 backbone stages were replaced by C3k2-UniRepLKNet modules. In the neck, the Feature Alignment Module aligned the P3, P4, and P5 feature maps, the Information Fusion Module produced a 256-channel global representation, and the injection modules redistributed the fused information to the three output levels. TADDH subsequently generated class predictions and bounding-box regression outputs at P3, P4, and P5. The principal architectural modifications are summarised in Table 2.

#### 2.3.2. UniRepLKNet Large-Kernel Convolutional Network

Given the dependence of dairy cow BCS on global morphological perception, the UniRepLKNet Block was introduced into the YOLOv11 backbone. Its large-kernel design expands the effective receptive field and can model spatial relationships among the hip bones, pin bones and tailhead more broadly than conventional small-kernel convolutions. This design is intended to complement local texture extraction with representation of the overall hindquarter contour, which may benefit discrimination between adjacent BCS grades.

**Figure 4:**
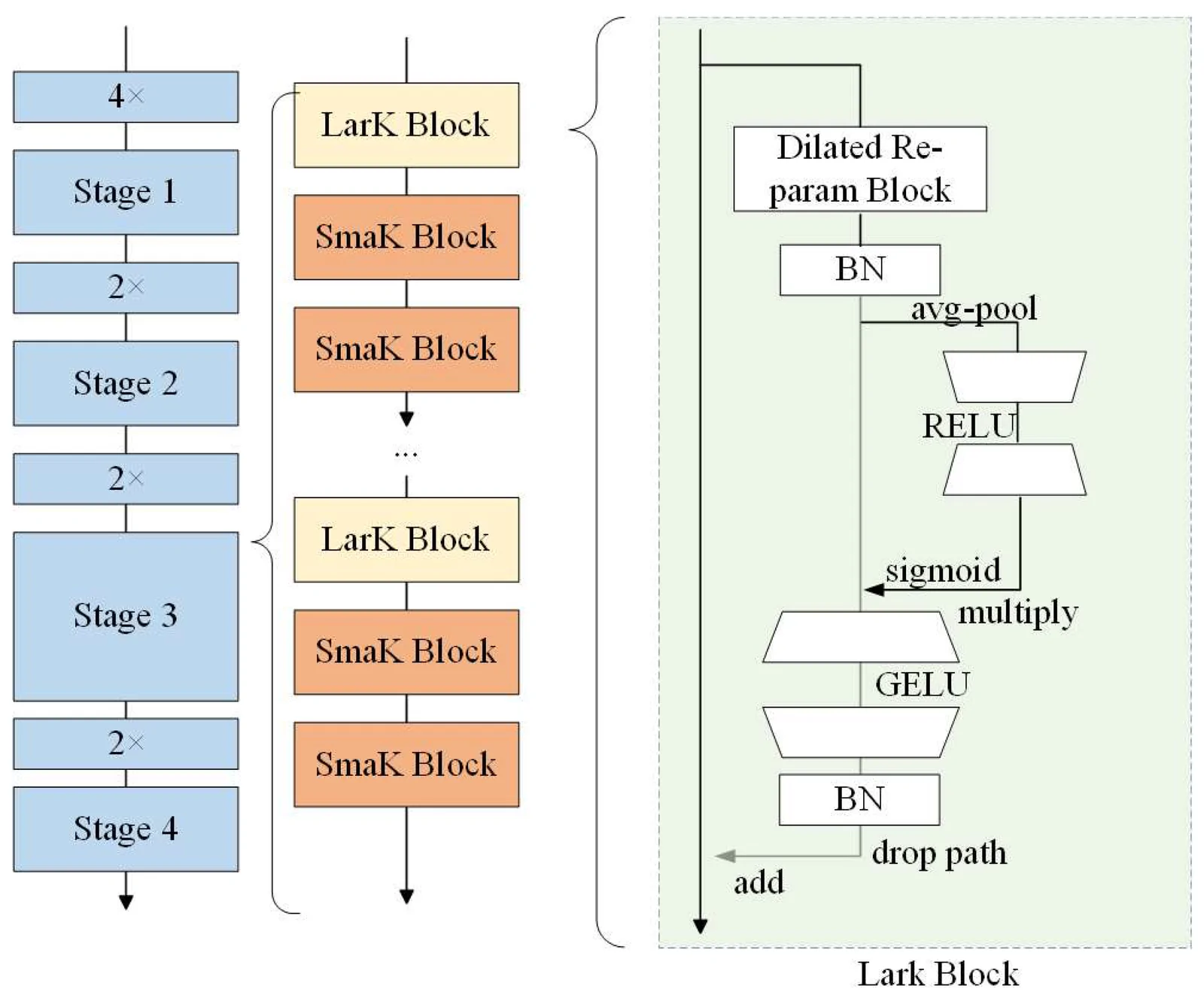
Structure diagram of UniRepLKNet.

UniRepLKNet uses structural re-parameterisation to support large-kernel optimisation. During training, auxiliary small-kernel branches facilitate optimisation; during inference, the parallel branches can be equivalently fused into a single convolutional operator. This design combines broad spatial modelling with a compact inference structure and preserves the convolutional inductive bias. In the present implementation, the C3k2-UniRepLKNet modules retained the input and output channel dimensions of the corresponding YOLOv11n C3k2 stages. Each UniRepLKNet unit consisted of a depthwise large-kernel convolution, batch normalisation, GELU activation, channel modulation, and a residual connection. During training, parallel dilated small-kernel branches were used to facilitate optimisation; before inference, the parallel branches were algebraically fused into a single large-kernel convolution. The modules were inserted at the P3/8, P4/16, and P5/32 backbone stages, with output channels of 64, 128, and 256, respectively. The structure used in this study is shown in Figure 4. In the present implementation, one UniRepLKNet unit was inserted within each C3k2-UniRepLKNet module at the P3/8, P4/16, and P5/32 stages. The principal depthwise convolution used a kernel size of 13 × 13. During training, parallel re-parameterisation branches used 5 × 5 convolutions with dilation rates of 1 and 2 and a 3 × 3 convolution with a dilation rate of 3. The channel expansion ratio was 0.5, and the drop-path rate was set to 0.0. The input and output channels remained 64, 128, and 256 at P3/8, P4/16, and P5/32, respectively. Before inference, the parallel convolutional and batch-normalisation branches were algebraically fused into a single 13 × 13 depthwise convolution.

#### 2.3.3. Gather-and-Distribute Feature Fusion Module

**Figure 5:**
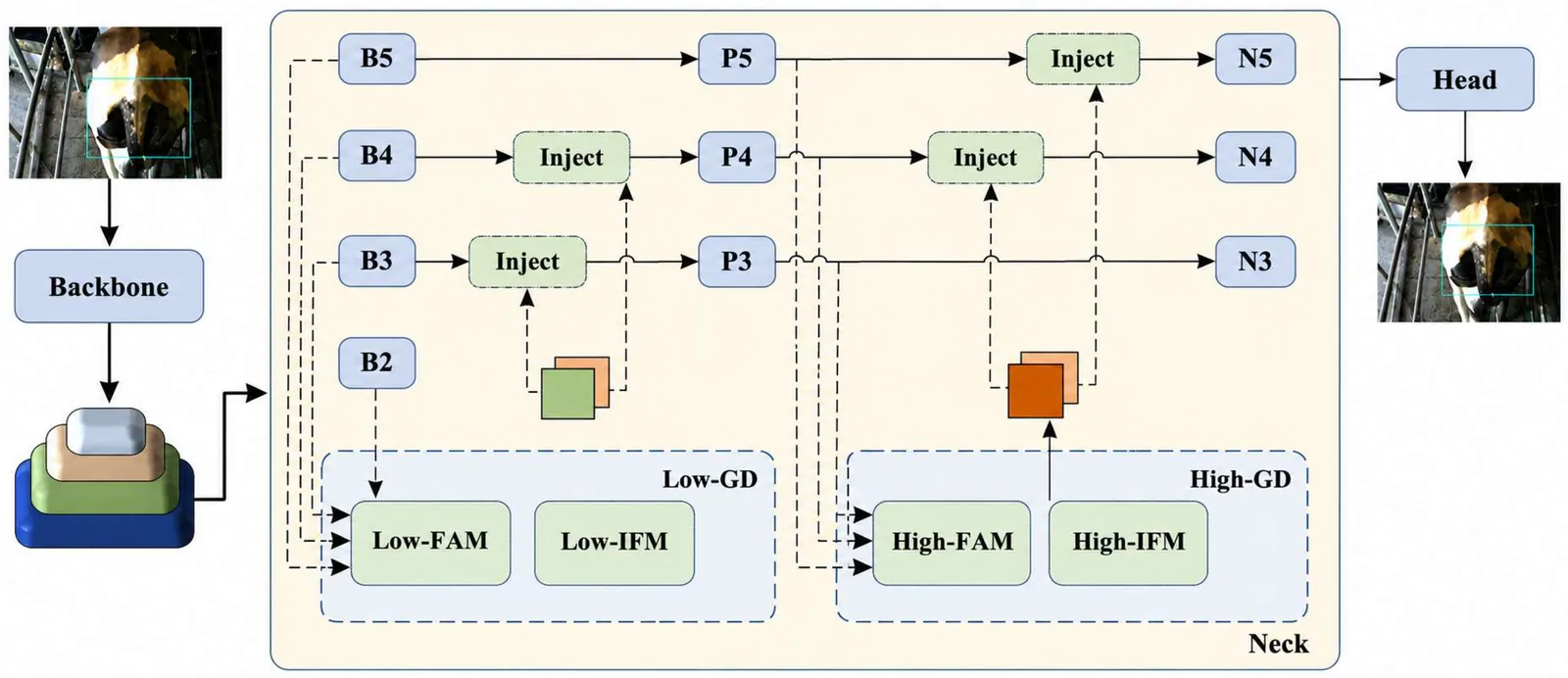
Structure diagram of the GD module.

The Gather-and-Distribute (GD) feature-fusion mechanism was adapted from Gold-YOLO to strengthen non-adjacent multiscale information interaction. The module received the P3, P4, and P5 backbone features with channel dimensions of 64, 128, and 256, respectively. The Feature Alignment Module first transformed the multiscale inputs to a common 256-channel representation and aligned their spatial dimensions through adaptive pooling or interpolation. The aligned features were concatenated and processed by the Information Fusion Module to generate an integrated global feature map. Injection modules then redistributed the fused representation to the P3, P4, and P5 branches through channel projection, local feature modulation, and residual fusion. This gather–fusion–redistribution process was designed to preserve fine bone-edge information from shallow features while introducing broader semantic context from deeper features. The GD structure is shown in Figure 5. The P3, P4, and P5 input features were first projected to 256 channels using 1 × 1 convolutions. P3 and P4 were aligned to a spatial resolution of 20 × 20 pixels using adaptive average pooling, whereas P5 was retained at its original 20 × 20 resolution. The three aligned features were concatenated to form a 768-channel tensor. The Information Fusion Module consisted of a 1 × 1 convolution reducing the concatenated tensor to 256 channels, followed by a 3 × 3 convolution with batch normalisation and SiLU activation. During redistribution, the global feature was resized to the target feature resolution using bilinear interpolation. Each injection module applied a 1 × 1 channel projection, sigmoid-based feature modulation, and residual addition to generate output features of 64, 128, and 256 channels for P3, P4, and P5, respectively.

#### 2.3.4. Task-Aligned Dynamic Detection Head

In dairy cow BCS, the visual features used for grade classification and the geometric features used for localisation may not be optimised at identical spatial positions. Although decoupled heads reduce direct interference between tasks, high classification confidence can still be associated with suboptimal localisation. To address this issue, TADDH combines task-interactive feature processing with task-specific prediction branches. Its alignment principle is based on TOOD, while its dynamic spatial and channel reweighting was informed by Dynamic Head.

**Figure 6:**
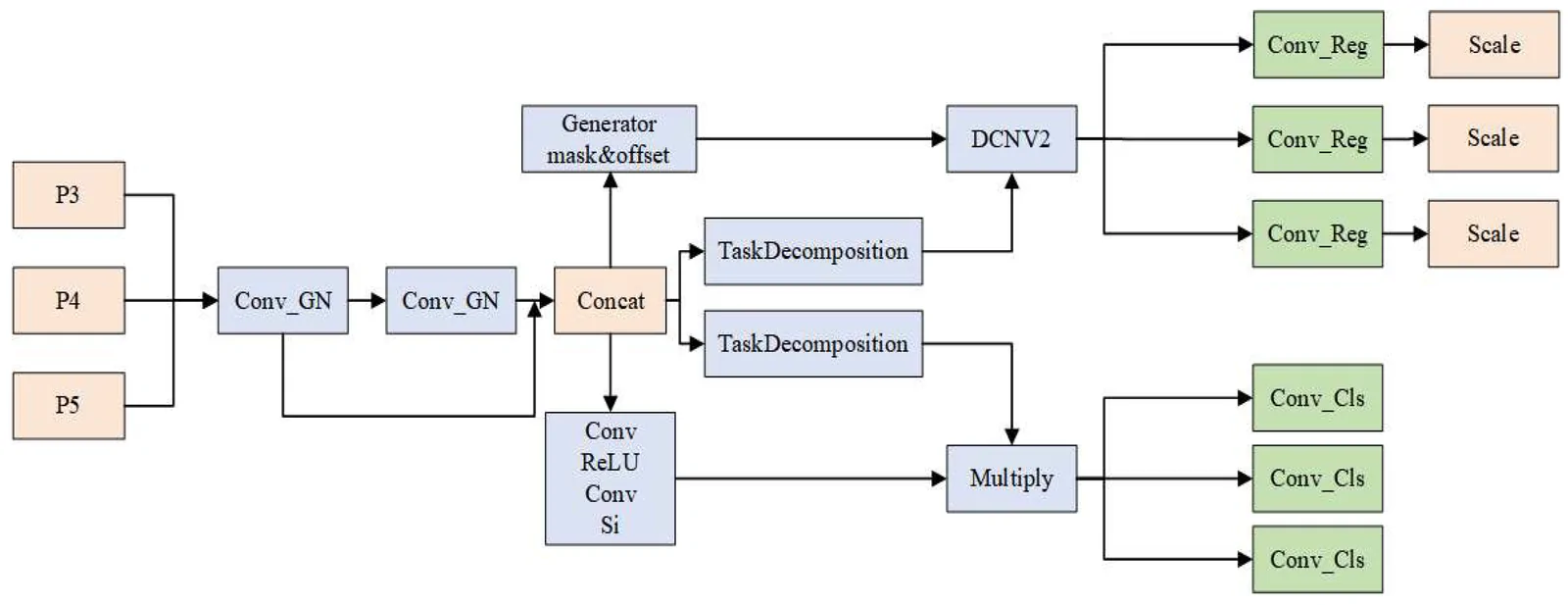
Structure diagram of TADDH.

The task-alignment metric is expressed as t = sα × IoUβ, where s is classification confidence, IoU represents localisation quality, and α and β are weighting exponents. The metric favours positive samples with jointly high classification and localisation quality. Dynamic feature modulation is then used to emphasise spatial regions and channels that are informative for the respective tasks. This formulation is intended to improve coordination between classification and regression rather than to treat them as fully independent objectives. The TADDH structure is shown in Figure 6.

In the present implementation, the task-alignment exponents were set to α = 0.5 and β = 6.0, and the top 10 candidate locations were retained during positive-sample assignment. Each prediction branch contained two 3 × 3 convolutional layers with 256 intermediate channels. The deformable convolution used a kernel size of 3 × 3, stride 1, padding 1, one convolution group, and one deformable group. Bounding-box regression used a distributional representation with reg_max = 16. The classification, bounding-box regression, and distribution focal loss gains were set to 0.5, 7.5, and 1.5, respectively.

### 2.4. Experimental Environment and Training Settings

#### 2.4.1. Hardware and Software Environment

All experiments were conducted using the hardware and software environment summarised in Table 3. YOLOv5s, YOLOv8n, YOLOv11n, and RT-DETR were implemented using the Ultralytics framework. SSD and Faster R-CNN were implemented using the torchvision detection framework, whereas DETR was implemented using its PyTorch-based architecture. UGT-YOLO was developed from the Ultralytics YOLOv11n implementation by integrating the UniRepLKNet Block, Gather-and-Distribute feature-fusion mechanism, and Task-Aligned Dynamic Detection Head. All models were trained and evaluated within the same Python and PyTorch environment.

#### 2.4.2. Training Parameters

Each comparison model was independently trained five times using random seeds of 0, 42, 3407, 2026, and 10,086. The training, validation, and test subsets remained fixed across all runs, whereas model initialisation, data-loader shuffling, and stochastic online augmentation varied according to the assigned random seed. For each run, the checkpoint achieving the highest validation mAP@0.5:0.95 was selected. The independent test set was evaluated only after model development and checkpoint selection had been completed. Performance metrics are reported as the mean ± standard deviation of the five independent runs. The detailed experimental model parameter settings are summarized in Table 4.

All models were trained from random initialisation. This design provided a controlled within-study comparison and avoided differences associated with pretrained-weight sources and compatibility. However, random initialisation may slow convergence and reduce absolute performance, particularly for architectures that are commonly trained using large-scale pretrained weights, such as DETR and Faster R-CNN. Therefore, the comparison results represent performance under the common experimental protocol used in this study rather than the independently optimised performance ceiling of each architecture.

The same cleaned dataset split, input resolution, evaluation procedure, and checkpoint-selection criterion were used for all comparison models. Architecture-specific network structures and native loss functions were retained. No additional detector-specific hyperparameter optimisation was performed. Consequently, the results should be interpreted as a controlled comparison under a common training protocol.

#### 2.4.3. Inference-Speed Evaluation

Inference speed was evaluated on an NVIDIA GeForce RTX 3090 using an input resolution of 640 × 640 pixels, a batch size of 1, and FP32 precision. Before formal timing, 100 warm-up iterations were performed to stabilise GPU execution and memory allocation. A total of 1000 consecutive inference iterations were subsequently timed, and the complete timing experiment was repeated five times.

CUDA synchronisation was performed immediately before and after each timed section. Image-file reading was excluded from the timing procedure, whereas deterministic image resizing, normalisation, network forward propagation, confidence filtering, and non-maximum suppression were included. FPS was calculated as the reciprocal of the mean end-to-end latency and is reported as the mean ± standard deviation of the five repeated timing experiments.

### 2.5. Evaluation Metrics

#### Ordinal BCS Evaluation

Because BCS is an ordinal variable, conventional object-detection metrics were supplemented with class-specific and ordinal evaluation metrics. Predicted boxes were matched one-to-one with ground-truth boxes using an IoU threshold of 0.50. For each matched ground-truth target, the predicted BCS category with the highest confidence was used for ordinal evaluation.

The five-class confusion matrix, macro-F1 score, and quadratic weighted Cohen’s kappa were calculated using the matched detections. Accuracy within ±0.25 and ±0.50 BCS units was calculated using all ground-truth targets, and an unmatched ground-truth target was counted as an incorrect prediction. Unmatched false-positive detections were reflected in precision, recall, AP, and mAP but were not included in the five-class confusion matrix.

Accuracy within ±0.25 BCS units was defined as the proportion of ground-truth targets for which the absolute difference between the predicted and reference BCS values was no greater than 0.25. Accuracy within ±0.50 BCS units, macro-averaged F1 score, and quadratic weighted Cohen’s kappa were also calculated. A five-class confusion matrix was constructed using the ordered categories 3.25, 3.50, 3.75, 4.00, and 4.25.

Precision (*P*) is a core metric for measuring the accuracy of classification predictions. Its physical meaning is the proportion of samples predicted as positive that are actually positive.

(1) $$P=\frac{\mathrm{TP}}{\mathrm{TP}+\mathrm{FP}}\;\times \;100\%$$

Recall (*R*) evaluates the model’s coverage ability for positive samples from a completeness perspective, representing the proportion of actual positive samples that are successfully predicted as positive.

To overcome the trade–off between precision and recall and to comprehensively evaluate model performance, Average Precision (*AP*) and mean Average Precision (mAP) are introduced as key metrics.

(2) $$R=\frac{\mathrm{TP}}{\mathrm{TP}+\mathrm{FN}}\;\times \;100\%$$

To account for the trade-off between precision and recall, average precision (*AP*) for class c at an intersection-over-union threshold τ was calculated as the area under the interpolated precision–recall curve:

(3) $$AP_{c}\left(\tau \right)=\int_{0}^{1}p_{interp,c}\left(r;\tau \right)\;dr$$

where r denotes recall, and $p_{interp,c}\left(r;\tau \right)$ denotes the interpolated precision for class c at recall r and IoU threshold τ. For a detection task containing C classes, mean average precision at a fixed IoU threshold was calculated as:

(4) $$mAP\left(\tau \right)=\frac{1}{C}\sum_{c=1}^{C}AP_{c}\left(\tau \right)$$

In the present study, C=5, corresponding to the BCS categories 3.25, 3.50, 3.75, 4.00, and 4.25. The metric mAP@0.5 represents the mean AP calculated at an IoU threshold of 0.50. The more stringent mAP@0.5:0.95 metric was calculated by averaging mAP over 10 IoU thresholds ranging from 0.50 to 0.95 in increments of 0.05:

(5) $$mAP@0.5:0.95=\frac{1}{10}\sum_{k=0}^{9}mAP\left(0.50+0.05k\right)$$

Therefore, mAP@0.5 primarily reflects detection performance under a relatively permissive localisation criterion, whereas mAP@0.5:0.95 provides a more comprehensive assessment of classification and localisation performance across increasingly stringent IoU thresholds.

## 3. Results

### 3.1. Comparison with Representative Object Detectors

To evaluate the performance of UGT-YOLO, it was compared with representative single-stage, two-stage, YOLO-based, and Transformer-based object detectors, including SSD, Faster R-CNN, YOLOv5s, YOLOv8n, YOLOv11n, DETR, and RT-DETR. All models were trained using the same fixed training, validation, and test subsets and were evaluated using the same test-set protocol. Each model was trained independently using five random seeds, and the reported precision, recall, mAP@0.5, and mAP@0.5:0.95 values represent the mean ± standard deviation across the five runs. Architecture-specific native loss functions were retained, but no model-specific hyperparameter optimisation was performed.

All comparison models were trained and evaluated using the same group-controlled training, validation, and test subsets. The input resolution was 640 × 640 pixels, the batch size was 32, and each model was trained for 200 epochs from randomly initialised weights. Architecture-specific detection heads, label-assignment procedures, and native loss formulations were retained because these components are intrinsic to the respective model designs. No architecture-specific hyperparameter optimisation was conducted. Consequently, the comparison represents performance under a common within-study protocol rather than the independently optimised maximum performance of each architecture. The comparative performance of UGT-YOLO and representative object detection models is presented in Table 5.

Values of P, R, mAP@0.5, and mAP@0.5:0.95 are presented as the mean ± standard deviation across five independent training runs. FPS is presented as the mean ± standard deviation across five repeated timing experiments. FLOPs, parameter counts, and model sizes are reported as single deterministic values.

### 3.2. Ablation Experiments

To evaluate the contribution of each proposed component, eight model variants, denoted A–H, were constructed based on YOLOv11n under the same experimental conditions, as shown in Table 6. Experiment A represents the baseline YOLOv11n model. Experiments B, C, and D independently incorporate UniRepLKNet, GD, and TADDH, respectively. Experiments E, F, and G evaluate pairwise combinations of the three components, whereas Experiment H integrates all three components.

A represents the baseline YOLOv11n model. B, C, and D contain UniRepLKNet, GD, and TADDH individually, respectively. E combines UniRepLKNet and GD; F combines GD and TADDH; G combines UniRepLKNet and TADDH; and H integrates all three proposed components. The corresponding ablation experiment results are presented in Table 7.

Among the individual modifications, Experiment B increased mAP@0.5 from 85.7% to 87.0%, while Experiments C and D achieved 86.4% and 86.2%, respectively. Combining UniRepLKNet and GD in Experiment E produced an mAP@0.5 of 88.1%. The complete model in Experiment H achieved the highest mAP@0.5 (88.5%) and mAP@0.5:0.95 (66.8%). Relative to the baseline, the complete model increased precision by 4.8 percentage points, recall by 0.9 percentage points and mAP@0.5 by 2.8 percentage points. The improvement was accompanied by increases in parameters and FLOPs and a reduction in throughput to 68 frames s^−1^.

**Figure 7:**
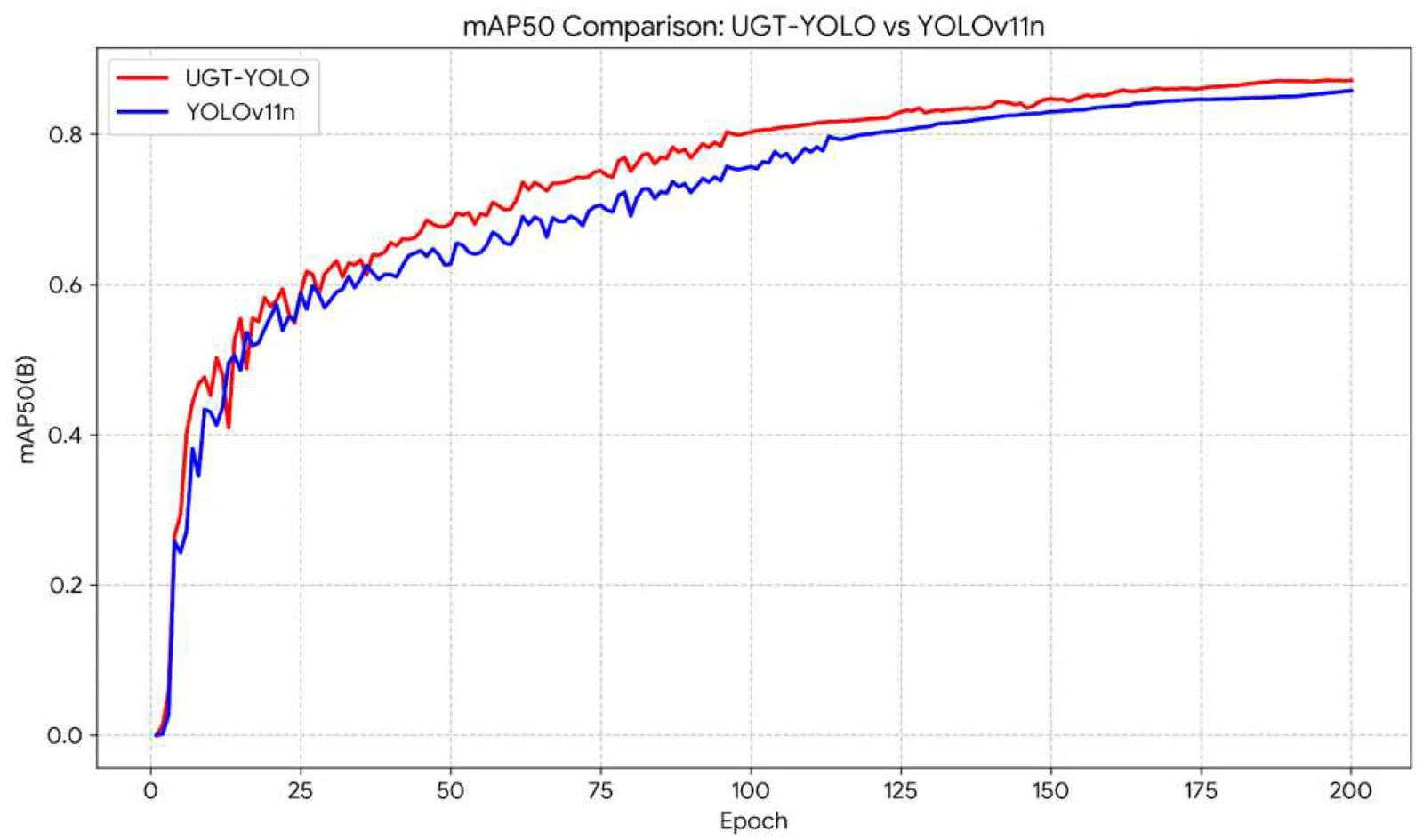
Validation mAP@0.5 curves of YOLOv11n and UGT-YOLO across 200 training epochs.

Figure 7 compares the validation mAP@0.5 curves of YOLOv11n and UGT-YOLO across 200 training epochs. The validation set was evaluated after each epoch and was used for checkpoint selection. The test set was not used to generate these curves and was evaluated only once after model development and checkpoint selection had been completed.

### 3.3. Class-Specific and Ordinal Evaluation

The class-specific results indicate that UGT-YOLO improved AP@0.5 across all five BCS categories. The largest improvement was observed for BCS 3.25, increasing from 82.6% to 86.0%, followed by BCS 4.25, which increased from 84.1% to 87.3%. These results suggest that the proposed model may provide particular benefits for the two boundary categories within the evaluated BCS range. The class-specific AP@0.5 results for YOLOv11n and UGT-YOLO are summarized in Table 8.

As shown in Table 9, the accuracy within ±0.25 BCS units increased from 76.6% for YOLOv11n to 78.9% for UGT-YOLO, while the accuracy within ±0.50 BCS units increased from 79.5% to 80.6%. The macro-F1 score increased from 0.798 to 0.827, and quadratic weighted kappa increased from 0.888 to 0.920. These results indicate that the improvement of UGT-YOLO was not limited to conventional detection metrics and may also improve agreement across the ordered BCS categories.

**Figure 8:**
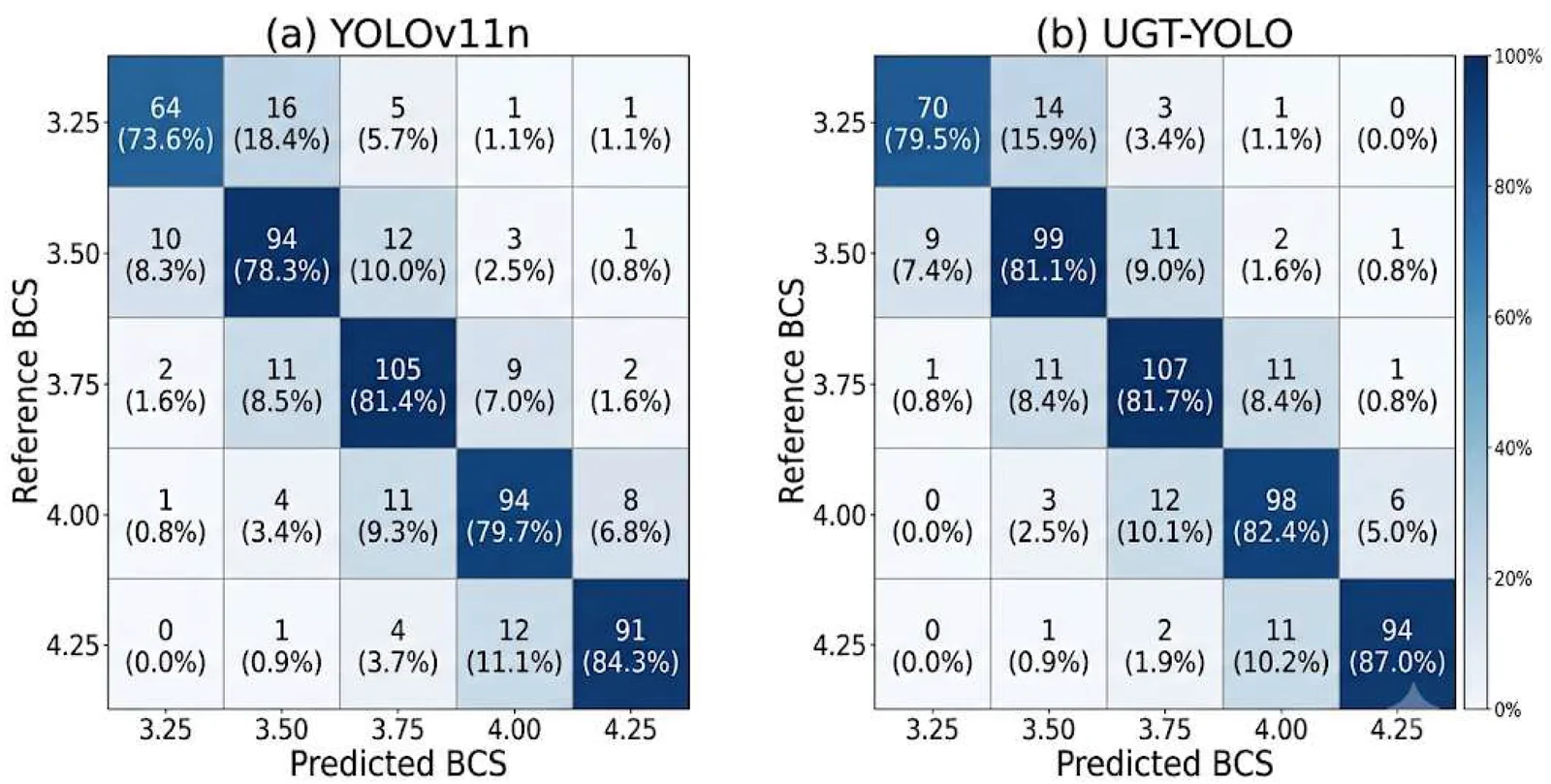
Row-normalised confusion matrices of (**a**) YOLOv11n and (**b**) UGT-YOLO on the test set.

To further examine the distribution of errors among the ordered BCS categories, the confusion matrices of YOLOv11n and UGT-YOLO are presented in Figure 8.

Most classification errors occurred between adjacent BCS categories. Compared with YOLOv11n, UGT-YOLO generally increased the diagonal proportions and reduced confusion between neighbouring categories, particularly for BCS 3.25 and 4.25.

Table 10 presents the class-specific effects of the proposed components. Compared with the baseline model in Experiment A, UniRepLKNet in Experiment B produced its largest AP@0.5 improvement for BCS 3.25, increasing AP@0.5 by 1.9 percentage points. The GD module in Experiment C produced its largest improvement for BCS 3.50, increasing AP@0.5 by 1.0 percentage point. TADDH in Experiment D produced its largest improvement for BCS 4.25, increasing AP@0.5 by 1.3 percentage points. The complete model in Experiment H achieved higher AP@0.5 than the baseline across all five BCS categories, with improvements of 3.4, 2.1, 2.5, 2.8, and 3.2 percentage points for BCS 3.25, 3.50, 3.75, 4.00, and 4.25, respectively.

## 4. Discussion

### 4.1. Comparison with Previous Automated BCS Studies

In the present study, UGT-YOLO achieved a precision of 84.3%, a recall of 81.1%, an mAP@0.5 of 88.5%, and an mAP@0.5:0.95 of 66.8%. Relative to YOLOv11n under the same experimental protocol, these values increased by 4.8, 0.9, 2.8, and 4.5 percentage points, respectively. The class-specific and ordinal results further indicated that the performance improvement was distributed across the five evaluated BCS categories rather than being limited to the overall mAP values.

Huang et al. [34] developed an improved YOLOv5s model for dairy cow body condition scoring and reported a precision of 93.4%, a recall of 85.5%, and an mAP@0.5 of 91.4%, together with a computational cost of 2.0 GFLOPs and a model size of 2.28 MB. Its reported mAP@0.5 was 2.9 percentage points higher than that of UGT-YOLO, while its computational cost and model size were substantially lower. However, the two studies differed in dataset processing, data partitioning, training configuration, and evaluation procedures. Consequently, the numerical difference should not be interpreted as a controlled head-to-head comparison. Dandıl et al. [35] applied YOLOv8x to Holstein and Simmental cow images collected from different farms. Their system correctly classified 102 of 126 test images, with an average classification accuracy of 0.81 and an average area under the precision–recall curve of 0.87. Although these metrics are not directly equivalent to object-detection mAP, the inclusion of two breeds, different farms, and BCS categories extending from emaciated to obese provides broader population and score-range coverage than the single public dataset and restricted BCS interval evaluated in the present study.

Zheng et al. [36] proposed YOLO-HGStar and reported a 16% reduction in model weight and a 3.4-percentage-point improvement in mAP relative to its corresponding baseline. By comparison, UGT-YOLO increased mAP@0.5 by 2.8 percentage points relative to YOLOv11n, but its parameter count increased from 2.6 to 6.5 million and its computational cost increased from 6.4 to 19.2 GFLOPs. The two studies therefore reflect different optimisation priorities: YOLO-HGStar emphasised lightweight improvement, whereas UGT-YOLO prioritised global morphological representation, cross-level feature interaction, and classification–localisation alignment. In addition to network architecture, the formulation of BCS categories can influence the apparent difficulty and performance of an automated scoring task. Nagy et al. [37] investigated the effects of score-class definition and annotation-region selection on deep learning-based BCS prediction. Their findings indicated that the score-class formulation should be considered when interpreting model performance, whereas changing the size of the annotated image region did not produce meaningful differences in prediction precision. This issue is particularly relevant to the present study because the five adjacent quarter-point categories contain substantially subtler morphological differences than broader grouped BCS categories.

Application-oriented studies have also evaluated outcomes that are not fully represented by image-level detection metrics. Albornoz et al. [38] evaluated a commercial three-dimensional camera system for repeated BCS measurement and reported that automated daily measurements improved precision and sensitivity for detecting within-cow changes over time compared with less frequent visual scoring. Their findings demonstrate the value of longitudinal evaluation, whereas the present study assessed independent images and did not investigate repeated BCS trajectories across lactation. Wang et al. [39] developed an Edge-IoT platform integrating dairy cow detection, individual identification, BCS estimation, and cloud-based information transmission. The system achieved an accuracy of 85% within a tolerance of ±0.50 BCS units, with a total inference time of 3.138 s per cow. In contrast, UGT-YOLO achieved an accuracy of 80.6% within ±0.50 BCS units, but its throughput was measured on an NVIDIA GeForce RTX 3090 rather than on a resource-constrained edge platform. Because the two studies used different sensors, datasets, system pipelines, and evaluation protocols, the numerical values should not be treated as a direct performance ranking. Nevertheless, the Edge-IoT study illustrates the additional system-level validation required for practical deployment.

More recently, Yao et al. [40] developed an automated regression-based BCS system using 3208 side-view images from 211 Holstein cows. Reference scores were independently assigned by three trained assessors, and stratified five-fold cow-level cross-validation was used to prevent images of the same cow from appearing in both training and validation subsets. Their optimal model achieved a mean absolute error of 0.41 and a Pearson correlation coefficient of 0.62. Explainability analysis further indicated that the model focused on anatomical regions such as the tailhead, hooks, pins, and ribs. Although regression metrics are not directly comparable with the detection metrics used in the present study, the multi-scorer reference labels, cow-level evaluation, and anatomical-region visualisation represent important methodological strengths. Matto et al. [41] compared manual reference scoring with two generations of commercial automated BCS systems. Both automated systems were associated with manual scores, but systematic differences remained. The older system tended to overestimate BCS, particularly in thinner cows, whereas the newer system showed closer agreement with manual assessment but still had limitations in detecting individual-level changes during early lactation. These results indicate that an automated system may be useful for herd-level management while still requiring further refinement for individual-animal monitoring. This distinction is important for UGT-YOLO because the present image-level results do not yet establish longitudinal accuracy or agreement with repeated manual scoring under commercial-farm conditions.

### 4.2. Interpretation of the Proposed Components and Computational Trade-Off

The ablation results indicate that the three proposed components made complementary contributions to model performance. Replacing the original backbone modules with C3k2-UniRepLKNet increased mAP@0.5 from 85.7% to 87.0%, representing the largest improvement produced by an individual component. This result is consistent with the hypothesis that a larger effective receptive field may improve representation of the overall hindquarter contour and broader spatial relationships among BCS-related anatomical structures.

The GD feature-fusion mechanism increased mAP@0.5 to 86.4% when used independently. Its contribution may be associated with improved interaction between shallow features containing local edge information and deeper features containing broader semantic information. TADDH increased mAP@0.5 to 86.2% and produced a positive improvement by coordinating classification confidence and localisation quality. Class-specific ablation results showed that UniRepLKNet produced its largest improvement for BCS 3.25, the GD module produced its largest improvement for BCS 3.50, and TADDH produced its largest improvement for BCS 4.25. When all three components were combined, the complete model achieved the highest mAP@0.5 and mAP@0.5:0.95, suggesting that global morphological representation, cross-level feature interaction, and task alignment address different aspects of fine-grained BCS detection.

The biological interpretation of these improvements should remain cautious. The current experiments demonstrate changes in detection performance but do not directly establish which anatomical regions contributed to individual predictions. Although the model design was motivated by structures such as the tailhead, hook bones, pin bones, and the overall hindquarter contour, the present study did not include feature visualisation or controlled anatomical-region occlusion analysis. Therefore, these anatomical interpretations should be regarded as architecture-based hypotheses rather than direct evidence of model attention. Future studies should combine feature visualisation with controlled occlusion experiments to quantify the contribution of individual anatomical landmarks.

The improvement in detection performance was accompanied by a clear increase in computational complexity. Relative to YOLOv11n, the parameter count increased from 2.6 million to 6.5 million, computational cost increased from 6.4 to 19.2 GFLOPs, and throughput decreased from 205 to 68 frames s^−1^. UGT-YOLO should therefore not be described as a strictly lightweight model. Instead, it provides an accuracy–efficiency trade-off and retains real-time image-level inference on an NVIDIA GeForce RTX 3090 under the reported benchmark conditions. Because inference speed depends on hardware, software, precision mode, preprocessing, and post-processing procedures, the reported FPS should not be directly extrapolated to embedded GPUs or other resource-constrained devices.

### 4.3. Ordinal Performance, Limitations, and Future Work

BCS is an ordinal variable, meaning that an error between adjacent quarter-point categories is less substantial than an error spanning several BCS intervals. Conventional object-detection metrics such as precision, recall, and mAP do not fully represent this ordered relationship. The present study therefore additionally reports class-specific AP, a five-class confusion matrix, macro-F1, quadratic weighted Cohen’s kappa, and accuracies within ±0.25 and ±0.50 BCS units.

The confusion matrices showed that most residual classification errors occurred between adjacent BCS categories. Compared with YOLOv11n, UGT-YOLO generally increased the diagonal proportions and reduced several confusions between neighbouring categories, particularly for BCS 3.25 and 4.25. The improvements in macro-F1, quadratic weighted kappa, and tolerance-based accuracy indicate that the performance gain was not limited to conventional detection metrics. Nevertheless, uncertainty in the reference labels should be considered because morphological differences between adjacent quarter-point categories can be subtle, and repeated scoring records and inter-rater agreement statistics were unavailable for the public dataset.

This study has several additional limitations. First, the dataset contained only five adjacent BCS categories ranging from 3.25 to 4.25. Performance for severely under-conditioned or over-conditioned cows cannot therefore be inferred, and the results should not be extrapolated to the complete 1–5 BCS scale.

Second, the study relied on the expert-assigned labels supplied with the public dataset and did not independently evaluate agreement with repeated manual scoring, multiple trained assessors, backfat thickness measurements, or other physiological reference measurements. Label uncertainty near adjacent class boundaries may consequently contribute to some apparent prediction errors.

Third, sequence-level independence was enforced during dataset partitioning, and all images originating from the same source sequence were assigned to a single subset. Cow-level independence was additionally enforced for records with reliable cow identifiers. However, complete cow identifiers were unavailable for all source sequences, and complete cow-level independence could therefore not be verified for records without reliable animal identifiers. Although the group-controlled partitioning procedure reduced the risk of leakage from temporally adjacent frames, independent cow-level validation remains necessary.

Fourth, no independent farm, breed, lactation-stage, or camera-specific dataset was used for external validation. The public dataset was obtained under a limited range of recording conditions, and model performance may be affected by changes in breed, coat pattern, posture, illumination, background, camera viewpoint, image quality, and farm-management environment. Prospective validation should therefore be conducted using independent cows and farms before practical deployment.

Fifth, all comparison models were trained from random initialisation. This strategy controlled differences associated with pretraining sources and weight compatibility, but it may have slowed convergence and reduced absolute performance, particularly for architectures commonly trained using large-scale pretrained weights. The comparison results should therefore be interpreted as performance under the common protocol used in this study rather than as the independently optimised performance ceiling of each architecture.

Finally, inference speed was evaluated using an RTX 3090 with a batch size of 1 and FP32 precision. This benchmark demonstrates real-time image-level inference under the reported laboratory conditions but does not represent performance on embedded or edge-computing devices. Future work should evaluate full-range BCS categories, independent cow-level and cross-farm datasets, repeated annotations from multiple scorers, continuous video processing, individual-animal identification, environmental robustness, model compression, and inference performance on practical edge hardware.

## 5. Conclusions

This study developed UGT-YOLO for five-class dairy cow body condition detection by integrating UniRepLKNet, a Gather-and-Distribute feature-fusion mechanism, and a Task-Aligned Dynamic Detection Head into YOLOv11n. On the single publicly available dataset evaluated in this study, UGT-YOLO achieved a precision of 84.3%, a recall of 81.1%, an mAP@0.5 of 88.5%, and an mAP@0.5:0.95 of 66.8%. Relative to YOLOv11n, these metrics increased by 4.8, 0.9, 2.8, and 4.5 percentage points, respectively.

The performance improvement was accompanied by an increase in parameters and computational cost and a reduction in inference throughput. Nevertheless, the model retained real-time image-level inference on an NVIDIA GeForce RTX 3090 under the reported benchmark conditions. These findings demonstrate an accuracy–complexity trade-off within the five evaluated BCS categories ranging from 3.25 to 4.25.

The conclusions are limited to the evaluated public dataset and restricted BCS range. Independent cow-level, cross-farm, full-range BCS, multi-scorer, and edge-device validation is required before the model can be considered suitable for practical deployment.

## Figures and Tables

**Table 1:** Class distribution before and after training-set augmentation.

BCS Class	Cleaned Original Total	Train Original	Offline Augmented Train	Final Train	Validation	Test	Final Total
3.25	1100	880	1050	1930	110	110	2150
3.50	1500	1200	725	1925	150	150	2225
3.75	1600	1280	648	1928	160	160	2248
4.00	1500	1200	728	1928	150	150	2228
4.25	1315	1052	876	1928	132	131	2191
Total	7015	5612	4027	9639	702	701	11,042

**Table 2:** Layer-level architectural configuration of UGT-YOLO.

Stage	Output Level	Spatial Size	Channels	Original Module	Modified Module
Backbone	P3/8	80 × 80	64	C3k2	C3k2-UniRepLKNet
Backbone	P4/16	40 × 40	128	C3k2	C3k2-UniRepLKNet
Backbone	P5/32	20 × 20	256	C3k2	C3k2-UniRepLKNet
Neck	P3/P4/P5	Multiscale	256 aligned	PAN/FPN	FAM
Neck	Global feature	20 × 20	256	PAN/FPN	IFM
Neck	P3/P4/P5	Multiscale	64/128/256	PAN/FPN	Injection modules
Head	P3/P4/P5	Multiscale	64/128/256	Detect	TADDH

**Table 3:** Experimental environment configuration.

Category	Item	Configuration
Hardware	GPU	NVIDIA GeForce RTX 3090, 24 GB VRAM
Hardware	CPU	Intel Xeon Gold 6330; 14 CPU cores allocated
Hardware	RAM	90 GB
Hardware	Storage	50 GB data disk
Software	Operating system	Ubuntu 22.04.5 LTS
Software	GPU driver	NVIDIA driver 570.124.04
Software	CUDA	CUDA 12.8
Software	cuDNN	cuDNN 9.7.1
Software	Python	Python 3.10.12
Software	Deep learning framework	PyTorch 2.7.1+cu128
Software	Computer-vision library	torchvision 0.22.1+cu128
Software	YOLO implementation	Ultralytics 8.3.0
Model implementation	YOLOv5s, YOLOv8n, YOLOv11n, and RT-DETR	Implemented using the Ultralytics framework
Model implementation	SSD and Faster R-CNN	Implemented using the PyTorch torchvision detection framework
Model implementation	DETR	Implemented using the official PyTorch-based DETR architecture
Model implementation	UGT-YOLO	Custom implementation developed from Ultralytics YOLOv11n by integrating UniRepLKNet, GD, and TADDH

**Table 4:** Experimental model parameter settings.

Parameter	Value	Description
Batch size	32	Number of training images processed per iteration
Input image size	640 × 640 pixels	Input images were resized to a fixed spatial resolution
Training epochs	200	Maximum number of complete training epochs
Workers	8	Number of CPU workers used for data loading
Optimiser	SGD	Stochastic gradient descent
Initial learning rate	0.010	Initial learning rate used for model optimisation
Momentum	0.937	Momentum coefficient of the SGD optimiser
Weight decay	0.0005	L2 regularisation coefficient
Learning-rate scheduler	Linear decay	Learning rate was progressively reduced during training
Final learning-rate factor	0.010	Ratio of the final learning rate to the initial learning rate
Warm-up epochs	3	Number of warm-up epochs
Mosaic closing epoch	Final 10 epochs	Mosaic augmentation was disabled during the last 10 epochs
Box regression loss	CIoU loss	Loss used for bounding-box regression
Classification loss	Binary cross-entropy loss	Loss used for BCS-category classification
Distribution focal loss	DFL	Distribution-based bounding-box regression loss
Box loss gain	7.5	Weight assigned to the box regression loss
Classification loss gain	0.5	Weight assigned to the classification loss
DFL loss gain	1.5	Weight assigned to the distribution focal loss
Random seeds	0, 42, 3407, 2026, and 10,086	Seeds used for the five independent training runs
Checkpoint-selection criterion	Highest validation mAP@0.5:0.95	Criterion used to select the best model checkpoint

**Table 5:** Comparison with representative object detection models.

Model	P	R	mAP@0.5	mAP@0.5:0.95	FLOPs (G)	Parameters (M)	Model Size (MB)	FPS
SSD	76.2 ± 0.7%	79.2 ± 0.8%	75.6 ± 0.6%	53.6 ± 0.7%	62.7	28.5	130.5	52.0 ± 0.8
YOLOv5s	78.6 ± 0.5%	78.4 ± 0.6%	82.1 ± 0.4%	58.3 ± 0.6%	4.2	2.3	5.3	145.0 ± 1.9
YOLOv8n	77.2 ± 0.6%	78.2 ± 0.6%	83.8 ± 0.4%	55.6 ± 0.7%	8.1	3.0	6.3	185.0 ± 2.4
YOLOv11n	79.5 ± 0.3%	80.2 ± 0.5%	85.7 ± 0.4%	62.3 ± 0.4%	6.4	2.6	5.5	205.0 ± 2.7
Faster R-CNN	76.1 ± 0.9%	81.2 ± 0.9%	68.0 ± 0.8%	48.8 ± 0.9%	270.2	137.1	169.1	18.0 ± 0.3
DETR	81.3 ± 0.8%	80.2 ± 0.9%	86.9 ± 0.6%	63.3 ± 0.8%	202.1	49.8	158.5	24.0 ± 0.4
RT-DETR	82.7 ± 0.6%	80.5 ± 0.7%	87.2 ± 0.5%	64.7 ± 0.6%	180.3	45.0	170.1	86.0 ± 1.1
UGT-YOLO	84.3 ± 0.4%	81.1 ± 0.5%	88.5 ± 0.4%	66.8 ± 0.5%	19.2	6.5	14.5	68.0 ± 0.9

**Table 6:** Ablation experiment design.

Component	A	B	C	D	E	F	G	H
+UniRepLKNet		√			√		√	√
+GD			√		√	√		√
+TADDH				√		√	√	√

**Table 7:** Ablation experiment results.

Experiment	P	R	mAP@0.5	mAP@0.5:0.95	FLOPs (G)	Parameters (M)	Model Size (MB)	FPS
A	79.5%	80.2%	85.7%	62.3%	6.4	2.6	5.5	205
B	81.2%	82.5%	87.0%	64.1%	15.0	5.5	12.4	88
C	80.4%	79.2%	86.4%	63.4%	12.2	4.8	10.7	102
D	80.1%	81.5%	86.2%	63.0%	12.0	4.8	11.7	105
E	82.8%	81.2%	88.1%	65.8%	17.8	6.1	13.6	75
F	83.1%	80.8%	87.4%	65.2%	13.8	5.2	11.8	85
G	82.2%	81.3%	87.2%	65.1%	16.5	5.7	12.9	80
H	84.3%	81.1%	88.5%	66.8%	19.2	6.5	14.5	68

**Table 8:** Class-specific AP@0.5 results of YOLOv11n and UGT-YOLO on the test set.

Model	BCS 3.25	BCS 3.50	BCS 3.75	BCS 4.00	BCS 4.25	mAP@0.5
YOLOv11n	82.6%	86.4%	88.3%	87.1%	84.1%	85.7%
UGT-YOLO	86.0%	88.5%	90.8%	89.9%	87.3%	88.5%

**Table 9:** Ordinal evaluation results of YOLOv11n and UGT-YOLO on the test set.

Model	Accuracy Within ±0.25	Accuracy Within ±0.50	Macro-F1	Quadratic Weighted κ
YOLOv11n	76.6%	79.5%	0.798	0.888
UGT-YOLO	78.9%	80.6%	0.827	0.920

**Table 10:** Class-specific AP@0.5 results of the ablation experiments.

Experiment	BCS 3.25	BCS 3.50	BCS 3.75	BCS 4.00	BCS 4.25	mAP@0.5
A	82.6%	86.4%	88.3%	87.1%	84.1%	85.7%
B	84.5%	87.5%	89.2%	88.0%	85.8%	87.0%
C	83.2%	87.4%	88.7%	87.4%	85.3%	86.4%
D	83.1%	86.8%	88.5%	87.2%	85.4%	86.2%
E	85.4%	88.7%	90.1%	89.0%	87.3%	88.1%
F	84.2%	88.3%	89.6%	88.3%	86.6%	87.4%
G	84.8%	87.7%	89.4%	88.1%	86.0%	87.2%
H	86.0%	88.5%	90.8%	89.9%	87.3%	88.5%

## Data Availability

The source dataset used in this study is entitled “Dairy cow body condition score target detection data set”. The processed dataset-splitting information and additional results generated during the present study are available from the corresponding author upon reasonable request.

## Abbreviations

**Abbreviation**	**Full term**
AP	Average precision
BCS	Body condition score
BCS-YOLO	Body condition scoring YOLO
CNN	Convolutional neural network
DETR	Detection Transformer
FAM	Feature Alignment Module
FN	False negatives
FP	False positives
FPN	Feature pyramid network
FPS	Frames per second
GD	Gather-and-Distribute
GFLOPs	Giga floating-point operations
GPU	Graphics processing unit
IFM	Information Fusion Module
IoU	Intersection over union
mAP	Mean average precision
NEFA	Non-esterified fatty acids
OS	Operating system
PANet	Path aggregation network
PASCAL VOC	PASCAL Visual Object Classes
RAM	Random-access memory
R-CNN	Region-based convolutional neural network
RGB	Red–green–blue
RT-DETR	Real-Time Detection Transformer
SGD	Stochastic gradient descent
SSD	Single Shot MultiBox Detector
TADDH	Task-Aligned Dynamic Detection Head
TOOD	Task-Aligned One-Stage Object Detection
TP	True positives
UGT-YOLO	UniRepLKNet–Gather-and-Distribute–Task-Aligned YOLO
UniRepLKNet	Universal Perception Large-Kernel Convolutional Network

## References

[1] Bewley J.M., Schutz M.M. (2008). An interdisciplinary review of body condition scoring for dairy cattle. Prof. Anim. Sci..

[2] Gearhart M.A., Curtis C.R., Erb H.N., Smith R.D., Sniffen C.J., Chase L.E., Cooper M.D. (1990). Relationship of changes in condition score to cow health in Holsteins. J. Dairy Sci..

[3] Zhang F., Tang X.F., Xiong B.H. (2025). Research progress on incidence patterns and prevention and control measures of ketosis in periparturient dairy cows. Chin. J. Anim. Nutr..

[4] Chen X.Y. (2025). Effects of Heat Adaptability During the Dry Period on Plasma Metabolism, Placental Function, and Calf Immunity in Dairy Cows. Master’s Thesis.

[5] Wang J., Zhang C., Zhao Q.Y., Jin S., Li C.C., Ma H., Liu D.K., Gu X.H. (2020). Effects of body condition score at dry off on calving performance, subsequent milk performance and incidence of disease in Holstein cows. Chin. J. Anim. Nutr..

[6] Wildman E.E., Jones G.M., Wagner P.E., Boman R.L., Troutt H.F., Lesch T.N. (1982). A dairy cow body condition scoring system and its relationship to selected production characteristics. J. Dairy Sci..

[7] Ferguson J.D., Galligan D.T., Thomsen N. (1994). Principal descriptors of body condition score in Holstein cows. J. Dairy Sci..

[8] Wu Y.F., Li Y.M., Zhao Y.Y., Yang P., Li Z.B., Guo H. (2021). Review of research on body condition score for dairy cows based on computer vision. Trans. Chin. Soc. Agric. Mach..

[9] Fischer A., Luginbühl T., Delattre L., Delouard J.M., Faverdin P. (2015). Rear shape in three dimensions summarized by principal component analysis is a good predictor of body condition score in Holstein dairy cows. J. Dairy Sci..

[10] Azzaro G., Caccamo M., Ferguson J.D., Battiato S., Farinella G.M., Guarnera G.C., Puglisi G., Petriglieri R., Licitra G. (2011). Objective estimation of body condition score by modeling cow body shape from digital images. J. Dairy Sci..

[11] Halachmi I., Polak P., Roberts D.J., Klopcic M. (2008). Cow body shape and automation of condition scoring. J. Dairy Sci..

[12] de Oliveira F.M., Ferraz P.F.P., Ferraz G.A.e.S., Pereira M.N., Barbari M., Rossi G. (2025). Prediction of body mass of dairy cattle using machine learning algorithms applied to morphological characteristics. Animals.

[13] Spoliansky R., Edan Y., Parmet Y., Halachmi I. (2016). Development of automatic body condition scoring using a low-cost 3-dimensional Kinect camera. J. Dairy Sci..

[14] Summerfield G.I., De Freitas A., van Marle-Koster E., Myburgh H.C. (2023). Automated cow body condition scoring using multiple 3D cameras and convolutional neural networks. Sensors.

[15] Shi W., Dai B., Shen W., Sun Y., Zhao K., Zhang Y. (2023). Automatic estimation of dairy cow body condition score based on attention-guided 3D point cloud feature extraction. Comput. Electron. Agric..

[16] Rodríguez Alvarez J., Arroqui M., Mangudo P., Toloza J., Jatip D., Rodríguez J.M., Teyseyre A., Sanz C., Zunino A., Machado C. (2018). Body condition estimation on cows from depth images using convolutional neural networks. Comput. Electron. Agric..

[17] Hansen M.F., Smith M.L., Smith L.N., Abdul Jabbar K., Forbes D. (2018). Automated monitoring of dairy cow body condition, mobility and weight using a single 3D video capture device. Comput. Ind..

[18] Liu D., He D., Norton T. (2020). Automatic estimation of dairy cattle body condition score from depth image using ensemble model. Biosyst. Eng..

[19] Martins B.M., Mendes A.L.C., Silva L.F., Moreira T.R., Costa J.H.C., Rotta P.P., Chizzotti M.L., Marcondes M.I. (2020). Estimating body weight, body condition score, and type traits in dairy cows using three-dimensional cameras and manual body measurements. Livest. Sci..

[20] Huang X., Hu Z., Wang X., Yang X., Zhang J., Shi D. (2019). An improved Single Shot MultiBox Detector method applied in body condition score for dairy cows. Animals.

[21] Li X.R. (2020). Research on Methods for Dairy Cow Body Condition Scoring Based on Deep Learning. Master’s Thesis.

[22] Sun Y., Huo P., Wang Y., Cui Z., Li Y., Dai B., Li R., Zhang Y. (2019). Automatic monitoring system for individual dairy cows based on a deep learning framework that provides identification via body parts and estimation of body condition score. J. Dairy Sci..

[23] Siachos N., Lennox M., Anagnostopoulos A., Griffiths B.E., Neary J.M., Smith R.F., Oikonomou G. (2024). Development and validation of a fully automated 2-dimensional imaging system generating body condition scores for dairy cows using machine learning. J. Dairy Sci..

[24] Mullins I.L., Truman C.M., Campler M.R., Bewley J.M., Costa J.H.C. (2019). Validation of a commercial automated body condition scoring system on a commercial dairy farm. Animals.

[25] Zheng Z., Wang Z., Weng Z. (2024). Efficient cow body condition scoring using BCS-YOLO: A lightweight, knowledge distillation-based method. Animals.

[26] Li J., Zeng P., Yue S., Zheng Z., Qin L., Song H. (2025). Automatic body condition scoring system for dairy cows in group state based on improved YOLOv5 and video analysis. Artif. Intell. Agric..

[27] Lewis R., Kostermans T., Brovold J.W., Laique T., Ocepek M. (2025). Automated body condition scoring in dairy cows using 2D imaging and deep learning. AgriEngineering.

[28] Liu F., Zhang Y., Liu Y., Li J., Li M., Yao J. (2025). A novel lightweight dairy cattle body condition scoring model for edge devices based on tail features and attention mechanisms. Vet. Sci..

[29] Huang X., Dou Z., Huang F., Zheng H., Hou X., Wang C., Feng T., Rao Y. Dairy Cow Body Condition Score Target Detection Data Set, Version 3. Science Data Bank. https://cstr.cn/31253.11.sciencedb.16704.

[30] Ding X., Zhang Y., Ge Y., Zhao S., Song L., Yue X., Shan Y. UniRepLKNet: A universal perception large-kernel ConvNet for audio, video, point cloud, time-series and image recognition. Proceedings of the IEEE/CVF Conference on Computer Vision and Pattern Recognition.

[31] Wang C., He W., Nie Y., Guo J., Liu C., Wang Y., Han K. Gold-YOLO: Efficient object detector via Gather-and-Distribute mechanism. Proceedings of the Advances in Neural Information Processing Systems 36, (NeurIPS 2023).

[32] Feng C., Zhong Y., Gao Y., Scott M.R., Huang W. TOOD: Task-Aligned One-Stage Object Detection. Proceedings of the IEEE/CVF International Conference on Computer Vision.

[33] Dai X., Chen Y., Xiao B., Chen D., Liu M., Yuan L., Zhang L. Dynamic Head: Unifying object detection heads with attentions. Proceedings of the IEEE/CVF Conference on Computer Vision and Pattern Recognition.

[34] Huang X., Feng T., Guo Y., Liang D. (2023). Lightweight dairy cow body condition scoring method based on improved YOLO v5s. Trans. Chin. Soc. Agric. Mach..

[35] Dandıl E., Çevik K.K., Boğa M. (2024). Automated classification system based on YOLO architecture for body condition score in dairy cows. Vet. Sci..

[36] Zheng Z., Wang Z., Weng Z., Poon T.-C., Jiang X., Wang Z., Tian J. (2025). Optimizing dairy cow condition scoring with YOLO-HGStar: A lightweight deep learning model. Proceedings of the Seventeenth International Conference on Digital Image Processing (ICDIP 2025), Haikou, China, 25–27 April 2025.

[37] Nagy S.Á., Kilim O., Csabai I., Gábor G., Solymosi N. (2023). Impact evaluation of score classes and annotation regions in deep learning-based dairy cow body condition prediction. Animals.

[38] Albornoz R.I., Giri K., Hannah M.C., Wales W.J. (2022). An improved approach to automated measurement of body condition score in dairy cows using a three-dimensional camera system. Animals.

[39] Wang J., Dai B., Li Y., He Y., Sun Y., Shen W. (2024). An intelligent Edge-IoT platform with deep learning for body condition scoring of dairy cow. IEEE Internet Things J..

[40] Yao L., Kong F., Hong W., Liu J., Li X., Fan Z.P., Lei L. (2026). Automated dairy cattle body condition score using side-view images and deep learning. J. Dairy Sci..

[41] Matto A., Ling K., Jaakson H., Teder S., Karis P. (2026). Comparison of manual gold standard with 2 automatic body condition scoring systems for dairy cows. JDS Commun..

